# Three Dimensional Printing Bilayer Membrane Scaffold Promotes Wound Healing

**DOI:** 10.3389/fbioe.2019.00348

**Published:** 2019-11-19

**Authors:** Shoubao Wang, Yao Xiong, Jingting Chen, Abdulsamad Ghanem, Yinmin Wang, Jun Yang, Binbin Sun

**Affiliations:** ^1^Department of Plastic and Reconstructive Surgery, Shanghai Ninth People's Hospital, Shanghai Jiao Tong University School of Medicine, Shanghai, China; ^2^Department of Orthopaedics, Shanghai Ninth People's Hospital, Shanghai Jiao Tong University School of Medicine, Shanghai, China

**Keywords:** three dimensional printing, bilayer membrane scaffold, wound healing, alginate, poly (lactic-co-glycolic acid)

## Abstract

Full-thickness skin wounds are common and could be a heavy physical and economic burden. With the development of three dimensional (3D) printing technology, skin-like constructs have been fabricated for skin wound healing and regeneration. Although the 3D printed skin has great potential and enormous advantages before vascular networks can be well-constructed, living cells are not recommended for 3D skin printing for *in vivo* applications. Herein, we designed and printed a bilayer membrane (BLM) scaffold consisting of an outer poly (lactic-co-glycolic acid) (PLGA) membrane and a lower alginate hydrogel layer, which respectively mimicked the skin epidermis and dermis. The multi-porous alginate hydrogel of the BLM scaffolds promoted cell adhesion and proliferation *in vitro*, while the PLGA membrane prevented bacterial invasion and maintained the moisture content of the hydrogel. Skin regeneration using the bilayer scaffold was compared with that of PLGA, alginate hydrogel and the untreated defect *in vivo*. Tissue samples were analyzed using histopathological and immunohistochemical staining of CD31. In addition, mRNA expression levels of collagen markers [collagen type 1 alpha 1 (COL1a1) and collagen type 3 alpha 1 (COL3a1)] and inflammatory markers [interleukin-1β (IL-1β), as well as tumor necrosis factor (TNF-α)] were measured. Conclusively, the application of BLM scaffold resulted in highest levels of best skin regeneration by increasing neovascularization and boosting collagen I/III deposition. Taken together, the 3D-printed BLM scaffolds can promote wound healing, and are highly suitable for a wide range of applications as wound dressings or skin substitutes.

## Introduction

Skin is the largest organ and the outermost protective sheath of the human body (Iii et al., 2000; Sun and Mao, 2012). It is highly susceptible to wounds, which are commonly caused by trauma, burns, skin diseases etc. Patients with full-thickness skin wounds suffer physical, psychological and economical burdens. Wound healing involves the coordination of many distinct but spatiotemporally overlapping physiological processes, including hemostasis, inflammation, epithelial cell proliferation, and tissue remodeling (Sabine and Richard, 2003; Eming et al., 2014; Sorg et al., 2017). Various natural and synthetic tissue engineering materials have been developed to accelerate wound healing, such as electrospun film, hydrogels, sponges etc. (Seo et al., 2012; Anisha et al., 2013; Dong et al., 2014; Catanzano et al., 2015; Zhao et al., 2017), which create a multi-porous and moist matrix that aids tissue regeneration. However, most scaffolds are composed of a single material, and therefore cannot simulate the functions of the full-thickness skin.

Three-dimensional printing is a personalized, flexible and accurate technology that is particularly suitable for wound healing (Murphy and Atala, 2014; Seol et al., 2014; Gu et al., 2015). Recent studies have reported *in vitro* fabrication of 3D simulated skin with dermis and epidermis-like structures through printing multiple layers of cells on various matrices (Lee et al., 2009, 2014; Skardal et al., 2012; Stefanie et al., 2013; Koch et al., 2015). In the absence of a vascular network, the 3D-printed skin relies solely on molecular diffusion and mechanical perfusion *in vivo*, which significantly reduces its viability since the diffusion range is generally limited to 100–200 μm (Tran and Wen, 2014;). Therefore, tissue construction by direct deposition or aggregation of living cells is not suitable for *in vivo* applications until microvascular networks can also be well-printed.

PLGA, the copolymer of poly (glycolic acid) and poly (lactic acid), is a biodegradable and biocompatible material that is used for tissue repair and reconstruction and drug delivery (Ueno et al., 2001; Danhier et al., 2012; Hudson and Margaritis, 2014). Since PLGA films are stiff, hydrophobic and semi-permeable, they do not have the capacity to absorb exudates or provide a moist microenvironment to accelerate wound healing (Ueno et al., 2001). These very characteristics however make PLGA a suitable outer layer that can separate a hydrogel matrix from the external environment, and retain the moisture content in the former. Furthermore, the nanofibers of the PLGA membrane are highly dense and can prevent bacterial invasion. Alginate, an anionic linear polysaccharide composed of (1,4)-linked β-D-mannuronic acid (M) and α-L-guluronic acid (G) blocks, is typically obtained from brown seaweed and is widely used in wound healing on account of its high histocompatibility, low toxicity, good bioresorption and low cost (Ueno et al., 2001; Boateng and Catanzano, 2015). It forms a hydrogel in the presence of divalent cations that cross-link with the G-blocks (Goh et al., 2012). Alginate hydrogels limit wound secretions and minimize bacterial contamination through super absorbance (Lee and Mooney, 2012). Furthermore, the multi-porous structure of these hydrogels promote cell invasion and neovascularization (Sun et al., 2018), and provide a physiologically moist microenvironment for wound healing (Boateng et al., 2014). Another major advantage of alginate hydrogel in the context of tissue reconstruction is the ease of 3D printing (Jahangir et al., 2018). Studies show that alginate wound dressings maintain a physiologically moist microenvironment, reduce bacterial infection at the wound site, and promote wound healing (Lee and Mooney, 2012).

**Figure 1:**
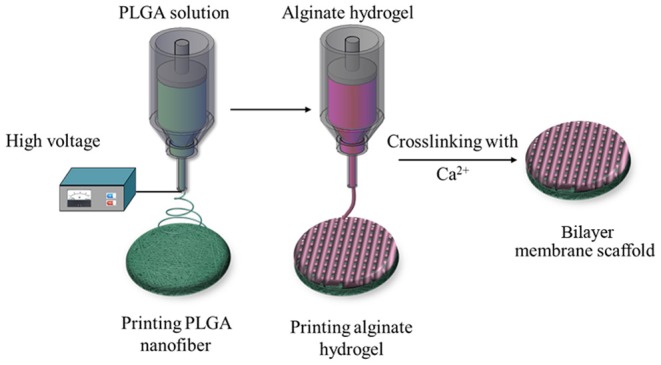
Schematic diagram. The schematic diagram of the bilayer membrane scaffold by 3D printing.

To this end, we used the 3D printing technology to create an BLM scaffold, with PLGA membrane as the superior layer and alginate membrane as inferior layer that, respectively, mimic the epidermis and dermis. Briefly, the first layer of PLGA nanofiber membrane was prepared using high voltage printing, while the second layer was fabricated by printing alginate hydrogel on the surface of PLGA nanofiber membrane. The schematic diagram of BLM fabrication is shown in Figure 1. The BLM scaffold provided an isolated and moist micro-environment that promoted inflammation, facilitated rapid vascularization and collagen deposition, and ultimately accelerated wound healing.

## Results and Discussion

### Characteristics of the BLM Scaffold

**Figure 2:**
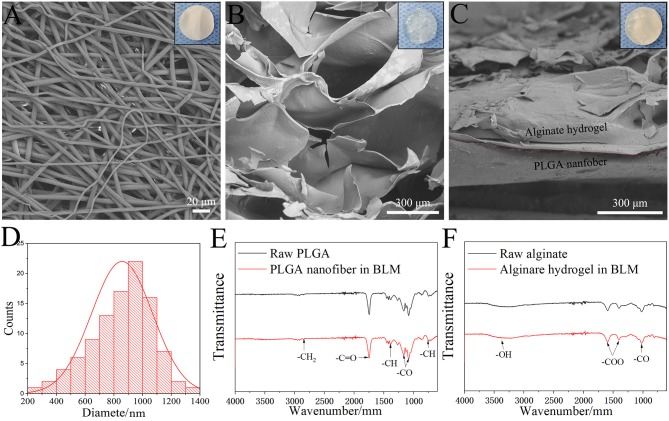
Morphology and structure. SEM image of PLGA nanofiber layer **(A)**, Alginate hydrogel layer **(B)** and BLM scaffold **(C)** (the top right-hand corner images are the corresponding digital photograph of each samples). **(D)** Nanofiber diameter distribution of PLGA nanofiber layer. FTIR results of raw PLGA and PLGA nanofiber in BLM **(E)**, and raw alginate and alginate hydrogel in BLM **(F)**.

**Figure 3:**
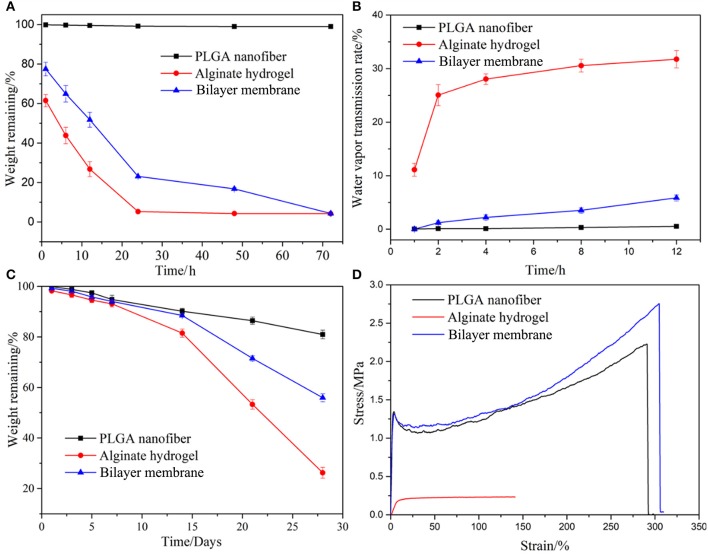
Properties characterization. The moisturizing **(A)**, water vapor transmission rate **(B)**, degradation **(C)**, and mechanical properties of PLGA nanofiber, alginate hydrogel and bilayer membrane scaffolds. **(D)** Stress-strain curves of three samples.

SEM images of the BLM scaffold revealed a dense outer layer of nano-sized PGLA fibers of diameter 857.02 ± 211.81 nm, and a loose microporous alginate hydrogel underneath. The thickness of the PLGA and alginate hydrogel layer were 20 and 100 μm, respectively. The FTIR results of the samples are shown in Figure 2. The spectrum of raw PLGA data shows absorbance peaks at wavelengths of 753 (CH-bend), 1,082 and 1,170 (CO stretch), 1,388 (CH-bend), 1,750 (CO ester), and 2,847 (CH_2_-bend) cm^−1^ (Figure 2E), while all spectrum peaks were observed in the PLGA nanofiber layer in BLM. Raw alginate produced a peak at 3,400 cm^−1^ indicating the presence of hydroxyl groups. Asymmetric and symmetric –COO groups are indicated by the peaks at a wavelength of 1,597 and 1,407 cm^−1^, respectively. The CO group is indicated by the peak at a wavelength of 1,027 cm^−1^. For alginate hydrogel layer in BLM, peaks were observed at the same sites (Figure 2F). The FTIR results showed that the preparation process does not affect the chemical properties of PLGA and alginate. The moisture retention and vapor transmission rates of the PLGA, alginate hydrogel and BLM scaffold are shown in Figure 3. While the hydrogel alone showed poor moisture retention and lost most of the water within 24 h, the BLM scaffold retained its moisture for 72 h (Figure 3A). Consistent with this, the PLGA nanofiber and BLM scaffolds had lower vapor transmission rates compared to that of alginate hydrogel (Figure 3B). Thus, the PLGA nanofiber membrane reduces evaporation from the alginate hydrogel. This is critical for wound healing since adequate moisture not only reduces the epithelial cell death but also promotes their migration and proliferation, and helps in tissue regeneration. Furthermore, while only 20% of the PLGA nanofiber degraded within 4 weeks, the degradation rate of alginate was slow in the first 2 weeks, and accelerated to 80% thereafter (Figure 3C), which is consistent with previous reports (Danhier et al., 2012; Lee and Mooney, 2012). The degradation rate of the BLM scaffold was intermediate to that of PLGA and alginate hydrogel. The rapid degradation of the hydrogel layer, which was designed to be in direct contact with the damaged area, is necessary for skin cell growth and tissue regeneration. In contrast, the outer PLGA membrane was designed to cover the wound for an extended period of time in order to prevent wound infection. The mechanical properties of the skin scaffold are critical for wound healing. The tensile stress, elongation at break, and Young's modulus of alginate were 231.51 ± 10.41 KPa, 139.18 ± 12.02% and 24.93 ± 2.45 KPa, respectively, which increased to 2753.58 ± 92.25 KPa, 304.28 ± 15.74% and 531.11 ± 12.64 KPa with the addition of the outer PGLA layer (Figure 3D). Taken together, the PLGA nanofiber layer has high tensile strength and can protect hydrogel while the latter aids in tissue regeneration.

### BLM Scaffold Resists Bacterial Invasion and Is Cyto-Compatible

**Figure 4:**
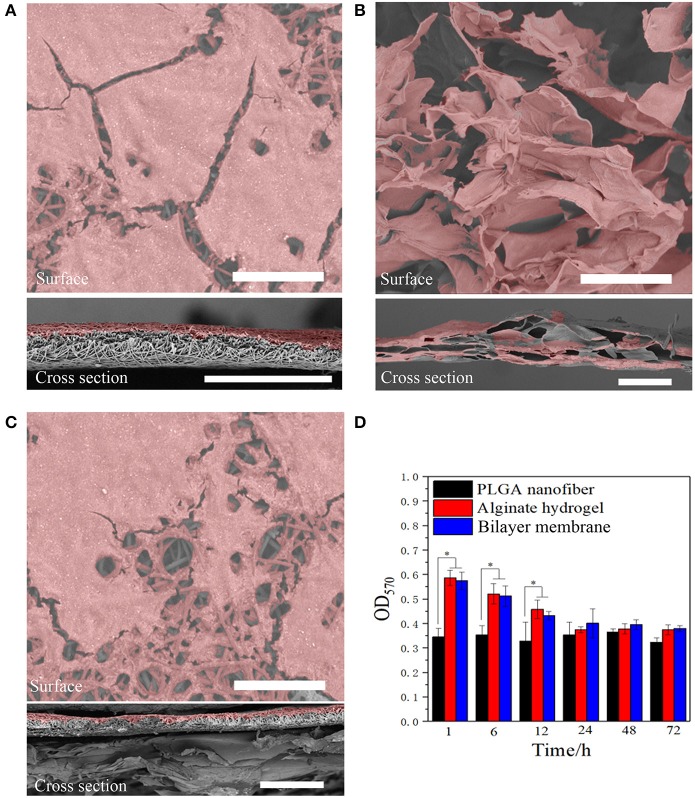
Anti-bacterial invasion and cell adhesion. SEM images of *Staphylococcus* bacterial on **(A)** PLGA nanofiber, **(B)** alginate hydrogel, and **(C)** bilayer membrane scaffold surface and cross section (the bacterial were labeled with pseudo-color, scare bar = 80 μm). **(D)** The cell adhesion results on three samples (*P < 0.05).

**Figure 5:**
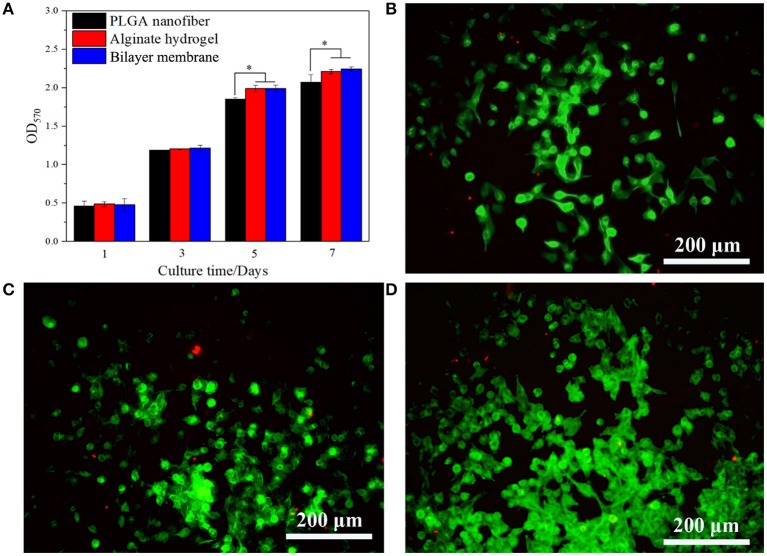
Cells viability and proliferation. **(A)** CCK-8 assay of L929 cells proliferation after incubation for 5 days on PLGA nanofiber, alginate hydrogel and bilayer membrane. **(B–D)** Live/Dead staining of L929 cells on PLGA nanofiber, alginate hydrogel and bilayer membrane on day 5. Living cells are labeled as green fluorescence, while dead cells are labeled as red fluorescence (*P < 0.05).

In addition to desirable mechanical properties and the ability to retain the moisture of the hydrogel, PGLA also acted as a barrier to bacterial invasion. As shown in Figures 4A–C, *S. aureus* was seeded onto the surface of three scaffolds. After the culture, it was found that the bacteria could infiltrate into the alginate hydrogel, but not into PLGA nanofiber or BLM scaffold. This indicates that the bacteria are able to penetrate into the hydrogel, but not into the PLGA layer, probably due to the high density nanofiber structure. This result provides evidence that the PLGA nanofiber layer can resist bacterial invasion. Furthermore, Figure 4D shows that murine fibroblasts are able to adhere to both the PLGA nanofibers and BLM scaffold regardless of their water content. In contrast, the alginate hydrogel and BLM scaffold had more cells adhered to them, compared with the PLGA nanofibers within the first 12 h. This demonstrates the stronger cell adhesion ability of alginate hydrogel, when it is moist. Therefore, it is essential to prevent water evaporation from the hydrogel to optimize cell adhesion during wound healing. In this regard, the BLM scaffold was more conducive to cell adhesion compared to pure hydrogel due to the protective PLGA layer. The biocompatibility of the different materials was tested by culturing murine L929 cells on the respective surfaces for 1, 3, 5, and 7 days. The cells spread and proliferated rapidly on all materials, indicating their biocompatibility (Figures 5A–D). However, after 5 and 7 days of incubation, the proliferative rate of the L929 cells growing on the alginate hydrogel and BLM scaffold were significantly higher compared to that cultured on the PLGA nanofibers, likely due to the higher biocompatibility, micro-porosity and water content of the hydrogel.

### BLM Scaffold Accelerated *in vivo* Wound Healing

**Figure 6:**
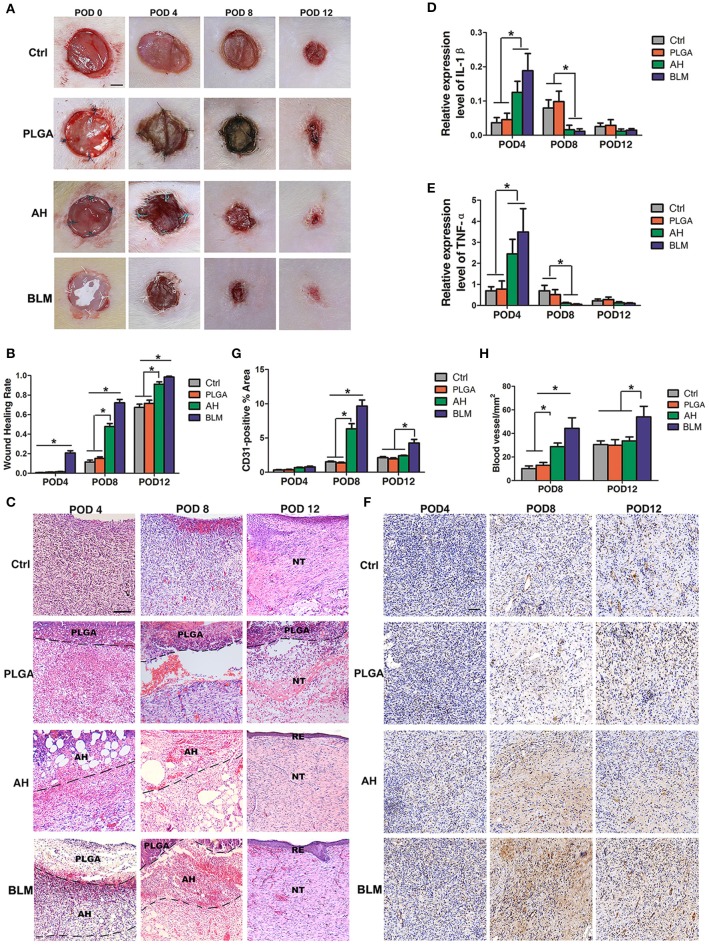
*In vivo* wound healing and staining. **(A)** *In vivo* wound healing of blank control, PLGA, alginate hydrogel (AH), and BLM scaffold on PODs 0, 4, 8, and 12; NT, new tissue; RE, re-epithelization (Scale bar 250 μm). **(B)** Analysis of wound healing rates in different groups. **(C)** H&E staining of control, PLGA, AH, and BLM scaffold on PODs 4, 8, and 12 (Scale bar 50 μm). **(D)** Q-PCR analysis of IL-1β in different groups on PODs 4, 8, and 12. **(E)** Q-PCR analysis of TNF-α in different groups on PODs 4, 8, and 12. **(F)** Immunohistochemical staining of different groups on PODs 4, 8, and 12 (Scale bar 5 μm). The red arrow indicate the vessels. **(G)** Quantification of blood vessel density in CD31-stained tissue sections. **(H)** Quantitative analysis of CD31-positive area (*P < 0.05).

*In vivo* wound healing was tested by implanting the respective biomaterials in full thickness wounds made in a rat model. All the animals survived, and showed gradual wound healing without infection, although the BLM scaffold induced the fastest wound closure. As shown in Figure 5A, the wound sizes were similar in the control, PLGA and alginate hydrogel groups after 4 days, whereas the BLM scaffold reduced wound size by 20.8% in the same time period (Figures 6A,B). On day 8 post wounding, the BLM and alginate hydrogel scaffolds resulted in 47.8 and 72.2% wound closure, respectively, whereas only 11.5 and 15.2% wound closure was observed in the control and PLGA groups (Figures 6A,B). The wounds treated with BLM scaffolds were completely healed by day 12, while 8.8, 28.5, and 33.7% of the wounds remained unhealed in the alginate hydrogel, PLGA and the control groups, respectively (Figures 6A,B). Histological examination revealed granulation tissue in the wound bed in all groups on the 4th day after the operation (Figure 6C), which was gradually replaced with new tissue in the BLM and alginate hydrogel-treated groups by day 8 (Figure 6C). On the 12th day, continuous and regular epithelial formation was seen on the alginate and BLM-treated wounds (Figure 6C), with a thicker epidermis thickness in the latter. In contrast, no continuous mature epithelial layer was seen in the control and PLGA groups. Taken together, the soft and porous structure of the alginate hydrogel accelerated wound healing by supporting cell adhesion, proliferation and migration (Lee and Mooney, 2012). In contrast, the hydrophobicity of PGLA prevents cell adhesion and migration (Danhier et al., 2012), which translated to poor wound healing in the absence of a supportive hydrogel layer.

### BLM Promotes Inflammation, Vascularization, and Collagen Deposition in the Wound

Wound healing is a complex process which is initiated with the clotting cascade and platelet activation, with the latter releasing pro-inflammatory cytokines like IL-1β and TNF-α. Senapati et al. and Ganeshkumar et al. showed that increased expression of pro-inflammatory cytokines in the early stages promoted wound healing (Senapati et al., 2011; Ganeshkumar et al., 2012) by recruiting leukocytes and activating fibroblasts, thereby promoting neovascularization and collagen deposition (Ganeshkumar et al., 2012). Consistent with this, IL-1β and TNF-α mRNAs were detected at all time points after surgery. The expression of IL-1β and TNF-α were significantly higher in the BLM and alginate hydrogel-treated groups compared to the PLGA and control groups on the 4th day (Figures 6D,E), and opposite trends were seen on the 8th and 12th day (Figures 6D,E).

Vascularization is critical for wound healing since that delivers nutrients and oxygen to the newly-formed tissues. In addition, neovascularization accelerates the migration of cellular and humoral factors to the wound (Eming et al., 2014), which in turn promote wound healing via formation of granulation tissue and collagen synthesis (Eming et al., 2014). We observed an increase in CD31-expressing endothelial cells in the BLM and alginate hydrogel-treated wounds on day 4 compared to the untreated control and PLGA-treated groups (Figures 6F,G). On day 8, the mean number of CD31+ cells in the wounds of the BLM, alginate hydrogel, PLGA and control groups were 9.66, 6.34, 1.36, and 1.5%, respectively (Figures 6F,G). Furthermore, the blood vessel density was significantly higher in the BLM and hydrogel-treated wounds compared to the untreated and PLGA-treated wounds (Figures 6F,H). The capacity of BLM and alginate hydrogel to stimulate neovascularization in the early stages of wound healing may be attributed to the 3D porous alginate scaffold. Interestingly, the CD31+ cells and blood vessel density was higher in the BLM-treated compared to the alginate hydrogel group on the 12th day after surgery (Figures 6F–H), possibly due to the PLGA membrane in the former which maintained the moist microenvironment of the hydrogel and accelerated neovascularization.

**Figure 7:**
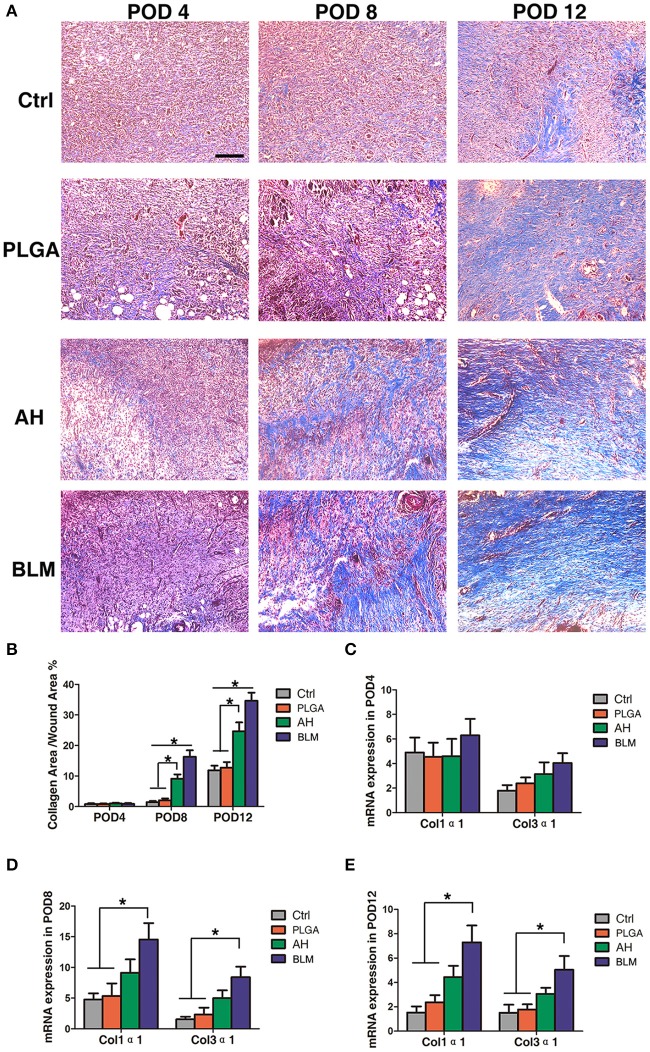
Collagen deposition. **(A)** Masson's staining of wounds in each group on PODs 4, 8, and 12 after operation. Scale bar, 5 μm. The red arrow indicates collagen fibers. **(B)** Quantification of trichrome blue stained area. **(C)** Q-PCR analysis of Col1α1 and Col3α1 in each group on POD4. **(D)** Q-PCR analysis of Col1α1 and Col3α1 in each group on POD8. **(E)** Q-PCR analysis of Col1α1 and Col3α1 in each group on POD12 (*P < 0.05).

Collagen synthesis and deposition play a vital role in wound healing by providing mechanical support to the tissues, and promoting cell adhesion, proliferation and differentiation (Eming et al., 2014). Masson's trichrome staining showed relatively sparse and disordered collagen fibers in the control and PLGA groups, while that in the BLM and alginate hydrogel groups were bundled and neatly arranged on day 4 (Figures 7A,B). With gradual wound healing, the deposition of collagen fibers increased in all groups. On the 8th and 12th days post wounding, the collagen fibers in the BLM and alginate hydrogel-treated groups were significantly denser compared to that in the control and PLGA groups (Figures 7A,B). Consistent with this, the relative expression of Col1α1 and Col3α1 were significantly higher in the BLM-treated wounds compared to the untreated and PLGA-treated wounds on days 8 and 12 (Figures 7C–E). Furthermore, the BLM-treated wounds had higher collagen content compared to the alginate-treated wounds (Figures 7A,B). The greater capacity of BLM scaffold to promote collagen deposition was likely related to the 3D porous alginate scaffold, as well as the outer protective PLGA membrane. Taken together, the BLM scaffold accelerates wound healing by promoting inflammation in the early stages, neovascularization and collagen synthesis.

## Conclusion

For this study, the BLM scaffold was created using 3D printing technology to mimic the skin structure, with PLGA nanofiber as the superior “epidermal” layer and alginate as the inferior “dermal” layer. The porous alginate hydrogel promoted cell adhesion and proliferation, compared with PLGA, whereas the BLM scaffold with a PLGA layer was able to prevent bacterial invasion and retain the humidity of the underlying hydrogel *in vitro*. Moreover, compared with the control, the PLGA and alginate hydrogel groups, the BLM scaffold displayed the strongest ability to promote inflammation, neovascularization and collagen I/III deposition, after implantation in the dorsal wound of rats, and ultimately accelerated wound healing. In conclusion, the above findings indicate that the 3D-printed BLM scaffold is optimal for wound healing *in vivo*. We found that the 3D-printed BLM scaffold is a highly promising type of wound dressing and skin substitute. Furthermore, the results obtained from this *in vivo* study can help make a thicker BLM scaffold that is suitable for use in prospective clinical studies on human wound healing.

### Materials

PLGA (Mw = 70–88 kDa, LA: GA = 50:50) was purchased from Jinan Daigang Co. Ltd. (China), and 1,1,1,3,3,3-Hexafluoro-2-propanol (HFIP) from Alfa Aesar Company (Ward Hill, MA, USA). Analytical grade sodium alginate and calcium chloride were obtained from Aladdin Co. Ltd (China).

### Fabrication of BLM Scaffold by 3D Printing

To prepare the PLGA nanofiber layer, the copolymer was dissolved in HFIP [w/v(%) = 15%] with constant stirring at room temperature for 4 h. The PLGA solution was placed in a barrel loaded in syringe pump and operated at a speed of 1.5 mL/h via the 3D printer (Tongli micro-nano technology Co., Ltd., Shenzhen, China). A high voltage of 12 kV was generated and connected to the printed needle site. The distance between the needle and the collector was 5–6 cm. The printing needle was set in a circular motion at the speed of 1 mm/s, and the layer height was set to 0.05 μm. PLGA nanofiber membranes of 10 mm diameter and 2 μm thickness were obtained.

The second hydrogel layer was prepared by dissolving sodium alginate in water with constant stirring at 40°C for 4 h. The hydrogel was placed in another barrel which was loaded in air compressor and operated at 0.05 MPa using the same 3D printer. The printing speed was 10 mm/s, and the layer height was set to 100 μm. The hydrogel layer was printed on the surface of the PLGA nanofibers membrane to obtain the BLM scaffold. The latter was then immersed into 5% (w/v) calcium chloride solution for 5 min to crosslink the alginate hydrogel, and then in sterile PBS for hydration. All 3D printing steps were performed under aseptic conditions at 25°C and 40% relative humidity. The equipment was UV-sterilized before 3D printing, and all biomaterials were sterilized through a 0.22 μm filter.

### Characterization of BLM Scaffold

The BLM scaffold was freeze-dried for 3 days, and its structure and surface morphology were observed under a scanning electron microscope (SEM, Hitachi TM-100, Japan) fitted with a digital camera (Cannon-50D, Japan). The raw PLGA, raw alginate, PLGA nanofiber layer in BLM scaffold, and alginate hydrogel layer in BLM scaffold were characterized using Fourier transform infrared spectroscopy (FTIR, Avatar 380 FTIR spectrometer). To analyze the moisture retention capacity, alginate hydrogel and BLM scaffold samples were weighed and dried at 37°C for 1, 6, 12, 24, 48, and 72 h in 24-well plates. The dried samples were weighed again, and the residual mass of samples was calculated according to the following formula:

(1) $$\begin{array}{l} \text{Weight remaining \%}=100\%\;\times \frac{W_{m}}{W_{i}} \end{array}$$

W_m_ is the mass of samples after evaporation (g), W_i_ is the initial mass of samples (g).

To determine the water vapor transmission rate, the pre-weighed samples were sealed and fixed on a utensil. The latter was then placed in a water-filled vessel and incubated at 37°C for 1, 2, 4, 8, and 12 h. The dried samples were weighed and water vapor transmission rate was calculated by the following formula:

(2) $$\begin{array}{l} \text{Water vapor trasmission rate \%}=100\%\;\times \frac{W_{t}}{W_{0}} \end{array}$$

W_t_ is the residual mass after evaporation (g), W_i_ is the initial mass (g).

The degradation of the alginate hydrogel, PLGA nanofiber and BLM scaffold *in vitro* was also measured by the loss of weight method. The pre-weighed samples were immersed in PBS containing 0.02 % (w/v) sodium azide and incubated at 37°C for 1, 3, 5, 7, 14, 21, and 28 days. The samples were weighed after the incubation period, and the residual mass (weight remaining %) of the samples degraded over time was calculated by the following formula:

(3) $$\begin{array}{l} \text{Weight remaining \%}=100\%\;\times \frac{W_{f}}{W_{i}} \end{array}$$

W_f_ is the mass of samples after degradation (g), W_i_ is the initial mass of samples (g). The above assays were repeated three times.

The mechanical strength of the samples (*n* = 5) was measured on a universal materials tester (HY-940FS, China) at room temperature and 60% humidity with stretching speed set at 10 mm per min. The stress-strain curve of each sample was observed, and the corresponding Young's modulus was calculated.

### Anti-bacterial Barrier and Mammalian Cell Support Function

BLM samples were fixed on nylon net supports that were then placed into petri dishes. Fifty microliters *Staphylococcus aureus* suspension containing 1 × 10^8^ colony-forming units (CFU)/ml was dropped on the center of each BLM sample, and the latter were immersed in culture medium. After incubating at 37°C for 24 h, the samples were fixed with 4% paraformaldehyde for 2 h, and dehydrated through an ethanol gradient (50, 70, 90, 95, and 100%). Bacterial invasion was observed by SEM, and the images were pseudo-colored using Photoshop CS 5.0.

To evaluate the surface adhesion of mammalian cells on the different materials, PLGA nanofiber, alginate hydrogel and BLM scaffold were dried for 1, 6, 12, 24, 48, and 72 h, and inoculated with 1 × 10^5^ murine fibroblast L929 cells. After 6 h of incubation, the non-adherent cells were removed with fresh medium, and then the number of adherent cells were counted using the Cell Counting Kit-8 (CCK-8 Beyotime, China). Cell proliferation was also analyzed on days 1, 3, 5 and 7 of culture. Briefly, 10 μL CCK-8 solution was added to each well and incubated for 2 h, and the absorbance was measured at 450 nm using a micro-plate reader. Viability was measured on day 5 using the LIVE/DEAD Viability/Cytotoxicity Kit (Life Technologies) according to the manufacturer's instructions.

### Establishment of Rat Dorsal Model of Wound Healing

Seventy-two 10-weeks old female Sprague-Dawley (SD) rats weighing 300–350 g were purchased from Xipu'er-bikai Experimental Animal Co. Ltd. (Shanghai, China). The animals were housed in specific pathogen-free conditions and had *ad libitum* access to food and water. All protocols were approved by the Animal Care and Use Committee of Shanghai Ninth People's Hospital Affiliated to Shanghai Jiaotong University School of Medicine approval no. SH9H-2019-A612-1. The rats were randomized into the untreated control, PLGA, alginate hydrogel and BLM scaffold groups (*n* = 18 each) prior to wound induction. Anesthesia in rats was induced using isoflurane, while the rats were in a chamber (isoflurane/oxygen: 5%).Thereafter the rats were placed in a prone position and allowed to breathe spontaneously through a nasal cone (isoflurane/oxygen: 1.5–2.0%). The backs of the rats were sterilized and shaved prior to surgery. Thereafter, three 10 mm biopsy punch wounds were made on each side of the dorsal midline, and the epidermis, dermis and perichondrium were removed to expose the underlying muscle tissue, as previously described previously (Gilmartin et al., 2016). The respective scaffolds were stitched to the edge of wounds using a 5-0 suture (Polypropylene suture (Ethicon U.S) for PLGA, Green Braided Polyester suture (Tevdek U.S) for alginate hydrogel and Gore-Tex suture (W.L. Gore & Associates U.S) for BLM, and the animals were placed on a 37°C heating plate for recovery.

### Wound Healing Evaluation

The wound area was measured on days 0, 4, 8, and 12 after operation (*n* = 6) using a sterile ruler, and the wound healing rate was calculated as the ratio of the areas of the healed and original wounds. Six animals from each group were sacrificed on days 4, 8, and 12 days after operation, and the wound tissue along with a 5 mm margin of surrounding intact tissue were removed. The tissues were fixed in 4% paraformaldehyde for 24 h and embedded in paraffin, sectioned, and stained with hematoxylin-eosin (H&E), Masson's trichrome and immuno-histochemical reagents (Song et al., 2018).

### Quantitative RT-PCR

Total RNA was extracted from the wound tissues, and reverse transcribed with Prime Script™ RT reagent Kit (RR037A, TaKaRa). Quantitative RT-PCR was performed using SYBR Green mixture on a QuantStudio™ 7 Flex Real-time PCR system (Applied Biosystems). The primers are listed in the table below.

**Table:** 

**Gene**	**Primer sequences**
IL-1β	Sense: 5′-CAGGCTTCGAGATGAACAACAA-3′ Antisense: 5′-ATCACTTGAGAGGTGGTCCCA-3′
TNF-α	Sense: 5′-CCCACGTCGTAGCAAACCACCA-3′ Antisense: 5′-CCATTGGCCAGGAGGGCGTTG-3′
Col1α1	Sense: 5′-CTCAAGATGTGCCACTCTGACT-3′ Antisense: 5′-GAGGGAGTTTACAGGAAGCAGAC-3′
Col3α1	Sense: 5′-TGGCGGCTTTTCACCATATT-3′ Antisense: 5′-ACTCTCTATTTGTCCGTTAACAGACTTG-3′
Gapdh	Sense: 5′-CCTTCATTGACCTCAACTAC-3′ Antisense: 5′-GGAAGGCCATGCCAGTGAGC-3′

### Statistical Analysis

Statistical analyses were performed using GraphPad Prism 5 software for Windows (GraphPad Software Inc., La Jolla, CA, USA), while comparison between multiple groups were made using single factor analysis of variance (ANOVA), and the differences between groups were evaluated using the Bonferroni *post-hoc* test.

## Data Availability Statement

The datasets generated for this study are available on request to the corresponding author.

## Ethics Statement

The animal study was reviewed and approved by animal care and use committee of shanghai ninth people's hospital affiliated to shanghai jiaotong university school of medicine.

## Author Contributions

SW designed the study and performed the experiments, analyzed the data and wrote the manuscript. YX performed the experiments and analyzed the data. AG wrote and reviewed the manuscript. YW and JC performed animal experiments. BS printed the bilayer membrane scaffold and characterized the properties of bilayer membrane scaffold. JY designed the study, discussed the data, and wrote and reviewed the manuscript.

### Conflict of Interest

The authors declare that the research was conducted in the absence of any commercial or financial relationships that could be construed as a potential conflict of interest.
